# Stability Analysis of a Delayed Rumor Propagation Model with Nonlinear Incidence Incorporating Impulsive Vaccination

**DOI:** 10.3390/e25121590

**Published:** 2023-11-27

**Authors:** Yuqian Zhou, Haijun Jiang, Xupeng Luo, Shuzhen Yu

**Affiliations:** 1 College of Mathematics and System Sciences, Xinjiang University, Urumqi 830017, China; 2 School of Mathematics and Statistics, Yili Normal University, Yining 835000, China; 3 School of Mathematics and Physics, Guangxi Minzu University, Nanning 530006, China; 4 School of Mathematics Science, Xinjiang Normal University, Urumqi 830017, China

**Keywords:** rumor propagation, impulsive vaccination, nonlinear incidence, time delay

## Abstract

The presence of information asymmetry can hinder the public’s ability to make well-informed decisions, resulting in unwarranted suspicion and the widespread dissemination of rumors. Therefore, it is crucial to provide individuals with consistent and dependable scientific education. Regular popular science education is considered a periodic impulsive intervention to mitigate the impact of information asymmetry and promote a more informed and discerning public. Drawing on these findings, this paper proposes a susceptible-hesitant-infected-refuting-recovered (SHIDR) rumor-spreading model to explain the spread of rumors. The model incorporates elements such as time delay, nonlinear incidence, and refuting individuals. Firstly, by applying the comparison theorem of an impulsive differential equation, we calculate two thresholds for rumor propagation. Additionally, we analyze the conditions of global attractiveness of the rumor-free periodic solution. Furthermore, we consider the condition for the rumor’s permanence. Finally, numerical simulations are conducted to validate the accuracy of our findings. The results suggest that increasing the proportion of impulsive vaccination, reducing the impulsive period, or prolonging the delay time can effectively suppress rumors.

## 1. Introduction

The widespread use of mobile communication and the rapid advancement of information technology have transformed online social networks (OSNs) such as Facebook, WeChat, and Twitter into vital platforms for information dissemination. However, along with this growth, the risk of rumor propagation on OSNs has also significantly increased. Rumors are unfounded statements lacking factual evidence that can create chaos and influence public opinions and actions. A tragic example of rumor-spreading is the belief that drinking can cure COVID-19, which has unfortunately led to the loss of hundreds of lives [1]. Therefore, it is crucial to comprehend the mechanisms of rumor-spreading and implement scientific interventions to mitigate the negative impact caused by the spread of rumors.

Considering the similarity between the spread of rumors and the transmission of epidemics, a significant number of existing models for rumor propagation draw inspiration from classical epidemic models. The DK model, introduced by Daley and Kendall in the 1960s, is widely recognized as the earliest description of rumor propagation models [2]. In the 1970s, Maki and Thompson further enhanced the DK model and introduced the MT model [3]. However, these models are primarily designed for analyzing the spread of rumors within small-scale social networks. With the emergence of social media platforms, rumors now have the ability to spread beyond traditional transmission methods. As a result, there has been a growing emphasis on studying the theory of rumor propagation in relation to the topological structure of social networks. Zanette conducted a study on the process of rumor-spreading in complex networks and found that the structure of the network plays a crucial role in the spread of rumors [4,5]. Moreno et al. applied the mean field theory to develop a rumor-spreading model specifically for heterogeneous networks [6]. This led to a growing interest in studying the dynamics of rumor propagation based on OSNs. In response to the challenges posed by rumor propagation, Liu et al. proposed a model that incorporates a dynamic quarantine strategy. This strategy effectively minimizes the negative impact on users’ daily routines while simultaneously curbing the spread of rumors [7]. During emergency situations, rumors tend to spread rapidly but eventually go through a cooling-off period [8]. Tong et al. considered the dynamics of rumor propagation with the presence of white noise disturbance in heterogeneous networks [9]. Furthermore, researchers have conducted studies on various factors to gain a comprehensive understanding of the mechanisms behind rumor propagation. These factors include group propagation [10], education [11], double-identity behavior [12], media coverage [13], individual activity and refutation mechanisms [14], hesitancy mechanisms [15], intervention mechanisms [16], and multilingual environments [17].

Indeed, when rumors circulate on OSNs, various stakeholders, including government agencies, typically collaborate to actively debunk false information and disseminate accurate information through the media. This concerted effort can significantly curtail the spread of rumors. Tian et al. proposed a model that takes into account the presence of both rumors and counter-rumors on OSNs [18]. Similarly, Pan et al. conducted a study on the impact of media coverage and individual refutation on the propagation of rumors. The findings revealed that strengthening the refutation process can serve as an effective strategy to control the spread of rumors [19]. Du et al. introduced a nonlinear SEIDR spreading model that takes into account the influence of social reinforcement and interest decay. Their findings suggest that it is valuable to incorporate debunkers and nonlinear functions into the analysis of rumor-spreading dynamics [20]. To fully comprehend the intricacies of rumor-spreading dynamics, it is essential to recognize the contribution of individuals who actively debunk rumors throughout the entire process.

The primary objective of studying the spread of rumors is to analyze the process of propagation in order to develop effective interventions that can mitigate any negative impact. Impulsive vaccination, which involves consistently providing accurate and comprehensive scientific education to the public, is an effective strategy for enhancing public knowledge and minimizing the circulation of misinformation. Cheng et al. [21] and Huo et al. [22] proposed rumor propagation models incorporating impulsive vaccination and time delays influenced by the media using bilinear incidence and standard incidence, respectively. Implementing impulsive vaccination at specific times can serve as a viable approach to controlling the spread of rumors. However, it is unfortunate that Cheng and Huo did not consider the influence of individuals who refute rumors. Therefore, it is crucial to study the effects of impulsive vaccination with an anti-rumor mechanism factor in order to gain valuable insights into the negative consequences of rumors on OSNs.

Many rumor models rely on the bilinear and standard incidence [23,24,25,26,27,28,29,30,31]. However, in reality, the number of effective contacts between individuals is limited due to factors such as crowding or precautions taken to prevent the spread of rumors, which can result in saturation at high transmission levels [32]. Furthermore, the spread of rumors is closely linked to the psychological characteristics of individuals. Therefore, it is more justifiable to incorporate psychological factors and utilize nonlinear incidence in the process of rumor propagation [16]. This approach allows for a more comprehensive depiction of the changes in individual attitudes and behaviors toward rumors. Additionally, time delays are a common occurrence in rumor-spreading, such as information updates. There is a time delay when individuals become aware of a rumor but do not take immediate action [33,34,35]. Hence, it is essential to take into account the time delay when susceptible individuals come into contact with affected or refuting individuals, as this influences their subsequent status as affected or refuting individuals.

To investigate the dynamics of rumor-spreading, an SHIDR model incorporating nonlinear incidence, refuting individuals, impulsive vaccination, and time delays is proposed. The key contributions of this study are as follows:

1. In contrast to previous studies [21,22], this research incorporates psychological factors and adopts a nonlinear incidence model, offering a more realistic depiction of the dynamics involved in rumor propagation.

2. This study incorporates popular science education as impulsive vaccination, aiming to enhance people’s knowledge and reduce the spread of rumors.

This paper is organized into seven sections. Section 2 presents an SHIDR rumor model that incorporates nonlinear incidence, refuting individuals, impulsive vaccination, and time delays. In Section 3, the existence of a rumor-free periodic solution is established. The global attractiveness of the rumor-free periodic solution is confirmed in Section 4. Section 5 discusses the system’s permanence. The theoretical analysis results are validated through numerical simulations in Section 6. Finally, the conclusions are presented in Section 7.

## 2. Rumor Propagation Model

Suppose that the total population is N(t), which can be divided into five distinct groups: susceptible individuals (S(t)), hesitant individuals (H(t)), infected individuals (I(t)), refuting individuals (D(t)), and recovered individuals (R(t)). Here, S(t) represents individuals who are susceptible to the rumors. H(t) denotes those who have heard the rumors but hesitate to either spread the rumor or spread the anti-rumor, I(t) indicates individuals who actively participate in spreading rumors, D(t) stands for those who do not believe in rumors and instead spread anti-rumors, and R(t) signifies individuals who neither believe nor spread any rumors. Susceptible individuals refer to individuals who have not yet been exposed to rumors but may come into contact with them. When susceptible individuals encounter rumors, they become hesitant individuals. In this state, they contemplate the veracity of the rumors and make a choice: either to believe and propagate the rumors, becoming infected individuals, or to disbelieve the rumors and disseminate the truth, becoming refuting individuals. In the presence of media reports, when infected individuals realize that the information they have transmitted is false, some of them will start spreading the truth and transition into refuting individuals, while others will choose not to propagate rumors or truth, becoming recovered individuals. For refuting individuals, there is also a certain probability that they will lose interest and cease spreading the truth, transitioning into recovered individuals. The parameters and their corresponding interpretations in Equation (1) are provided in Table 1.

When exposed to media reports, if the infected individuals become aware that the rumor information is false, then they will transform into refuting individuals with a probability of $\left(1-\theta \right)\gamma_{1}$. Alternatively, they will refrain from further spreading the rumor or spreading the anti-rumor with a probability of $\theta \gamma_{1}$. In a scenario where susceptible individuals come into contact with infected individuals and refuting individuals, the nonlinear incidences of the infected individuals and the refuting individuals can be represented by the terms $\alpha_{1}\frac{S(t)I(t)}{1+\rho_{1}S\left(t\right)}$ and $\alpha_{2}\frac{S(t)D(t)}{1+\rho_{2}S\left(t\right)}$, respectively. By disseminating popular science information, the government assists in enhancing the public’s understanding and acceptance of factual information through the media, with the aim of controlling the spread of rumors. Popular science education is often delivered periodically and with repetition, contributing to the regularity of its impact. Therefore, this paper argues that popular science education can be considered a periodic process akin to impulsive interventions, which have limited impact on the infected individuals. The assumption made in this paper is that impulsive vaccination only affects susceptible individuals. Based on these assumptions, an impulsive rumor propagation model is established as follows:

(1) $$\left\{\begin{array}{c} t\neq nT\left\{\begin{array}{c} \begin{array}{cc} \frac{dS(t)}{dt} & =\pi -\alpha_{1}\frac{S(t)I(t)}{1+\rho_{1}S\left(t\right)}-\alpha_{2}\frac{S(t)D(t)}{1+\rho_{2}S\left(t\right)}-\mu S\left(t\right),\\ \frac{dH(t)}{dt} & =\frac{\alpha_{1}S\left(t\right)I\left(t\right)}{1+\rho_{1}S\left(t\right)}+\frac{\alpha_{2}S\left(t\right)D\left(t\right)}{1+\rho_{2}S\left(t\right)}-\alpha_{1}e^{-\mu \tau }\frac{S(t-\tau )I(t-\tau )}{1+\rho_{1}S\left(t-\tau \right)}\\ & \;\;\;-\alpha_{2}e^{-\mu \tau }\frac{S(t-\tau )D(t-\tau )}{1+\rho_{2}S\left(t-\tau \right)}-\mu H\left(t\right),\\ \frac{dI(t)}{dt} & =ke^{-\mu \tau }S\left(t-\tau \right)\left(\frac{\alpha_{1}I\left(t-\tau \right)}{1+\rho_{1}S\left(t-\tau \right)}+\frac{\alpha_{2}D\left(t-\tau \right)}{1+\rho_{2}S\left(t-\tau \right)}\right)-\mu I\left(t\right)-\gamma_{1}I\left(t\right),\\ \frac{dD(t)}{dt} & =\left(1-k\right)e^{-\mu \tau }S\left(t-\tau \right)\left(\frac{\alpha_{1}I\left(t-\tau \right)}{1+\rho_{1}S\left(t-\tau \right)}+\frac{\alpha_{2}D\left(t-\tau \right)}{1+\rho_{2}S\left(t-\tau \right)}\right)\\ & \;\;\;-\gamma_{2}D\left(t\right)-\mu D\left(t\right)+\left(1-\theta \right)\gamma_{1}I\left(t\right),\\ \frac{dR(t)}{dt} & =\theta \gamma_{1}I\left(t\right)+\gamma_{2}D\left(t\right)-\mu R\left(t\right), \end{array} \end{array}\right.\\ t=nT\left\{\begin{array}{c} \begin{array}{cc} S\left(t^{+}\right) & =(1-\varphi )S(t),\\ H\left(t^{+}\right) & =H(t),\\ I\left(t^{+}\right) & =I(t),\\ D\left(t^{+}\right) & =D(t),\\ R\left(t^{+}\right) & =R(t)+\varphi S(t). \end{array} \end{array}\right. \end{array}\right.$$

**Figure 1:**
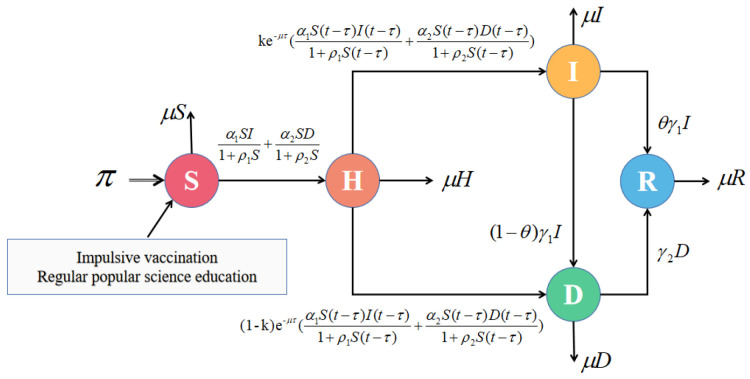
Structure of the process of rumor propagation.

An illustration of the rumor-spreading process can be seen in Figure 1. By taking the derivative of N(t) with respect to *t* using the system in Equation (1), we obtain the following expression:

$$\begin{array}{cc} \frac{dN(t)}{dt} & =\frac{dS(t)}{dt}+\frac{dH(t)}{dt}+\frac{dI(t)}{dt}+\frac{dD(t)}{dt}+\frac{dR(t)}{dt}\\ \; & =\pi -\mu N(t). \end{array}$$

Hence, we have

(2) $$\lim_{t\to \infty }N\left(t\right)=\frac{\pi }{\mu }.$$

Equation (1) is simplified as follows:

(3) $$\left\{\begin{array}{c} t\neq nT\left\{\begin{array}{c} \begin{array}{cc} \frac{dS(t)}{dt} & =\pi -\alpha_{1}\frac{S(t)I(t)}{1+\rho_{1}S\left(t\right)}-\alpha_{2}\frac{S(t)D(t)}{1+\rho_{2}S\left(t\right)}-\mu S\left(t\right),\\ \frac{dI(t)}{dt} & =ke^{-\mu \tau }S\left(t-\tau \right)\left(\frac{\alpha_{1}I\left(t-\tau \right)}{1+\rho_{1}S\left(t-\tau \right)}+\frac{\alpha_{2}D\left(t-\tau \right)}{1+\rho_{2}S\left(t-\tau \right)}\right)-\mu I\left(t\right)-\gamma_{1}I\left(t\right),\\ \frac{dD(t)}{dt} & =\left(1-k\right)e^{-\mu \tau }S\left(t-\tau \right)\left(\frac{\alpha_{1}I\left(t-\tau \right)}{1+\rho_{1}S\left(t-\tau \right)}+\frac{\alpha_{2}D\left(t-\tau \right)}{1+\rho_{2}S\left(t-\tau \right)}\right)\\ & \;\;\;-\gamma_{2}D\left(t\right)-\mu D\left(t\right)+\left(1-\theta \right)\gamma_{1}I\left(t\right),\\ \frac{dR(t)}{dt} & =\theta \gamma_{1}I\left(t\right)+\gamma_{2}D\left(t\right)-\mu R\left(t\right),\\ \frac{dN(t)}{dt} & =\pi -\mu N(t), \end{array} \end{array}\right.\\ t=nT\left\{\begin{array}{c} \begin{array}{cc} S\left(t^{+}\right) & =(1-\varphi )S(t),\\ I\left(t^{+}\right) & =I(t),\\ D\left(t^{+}\right) & =D(t),\\ R\left(t^{+}\right) & =R(t)+\varphi S(t),\\ N\left(t^{+}\right) & =N(t). \end{array} \end{array}\right. \end{array}\right.$$

The initial conditions for the system in Equation (3) are

(4) $$\begin{array}{cc} \; & S\left(\theta \right)=\xi_{1}\left(\theta \right),\;I\left(\theta \right)=\xi_{2}\left(\theta \right),\;D\left(\theta \right)=\xi_{3}\left(\theta \right),\;R\left(\theta \right)=\xi_{4}\left(\theta \right),\;N\left(\theta \right)=\xi_{5}\left(\theta \right),\\ \; & \xi \left(\theta \right)=\left(\xi_{1}\left(\theta \right),\;\xi_{2}\left(\theta \right),\;\xi_{3}\left(\theta \right),\;\xi_{4}\left(\theta \right),\;\xi_{5}\left(\theta \right)\right)\in C_{+},\;\xi_{i}\left(\theta \right)\geq 0, \end{array}$$

where$\;C_{+}=C\left(\left[-\tau ,\;0\right],\;R_{+}^{5}\right),\;R_{+}^{5}=\left\{\left(x_{1},\;x_{2},\;x_{3},\;x_{4},\;x_{5}\right)\in R^{5}:x_{i}⩾0,i=1,\;2,\;3,\;4,\;5\right\}.$

**Remark** **1.** *The solutions generated by the system in Equation *(3)* are non-negative and bounded for all t>0. The feasible region *Ω* is considered a positively invariant set of the system in Equation *(3)* with the initial conditions in Equation *(4)*, which are defined as follows:*

(5) $$\Omega =\left\{\left(S,I,D,R,N\right)\in R_{+}^{5}|0⩽S+I+D+R,\;N⩽\frac{\pi }{\mu }\right\}.$$

**Remark** **2.** *This paper introduces a fresh perspective on investigating the dissemination of rumors. In contrast to previous studies [21,22], this paper takes into account the influence of refuting individuals in the model. Additionally, it considers the effects of nonlinear incidence, offering valuable insights for the formulation of effective strategies to mitigate the proliferation of rumors.*

## 3. Existence of Rumor-Free Periodic Solutions

**Lemma** **1.** *The system in Equation *(3)* has the rumor-free periodic solution $\left({\tilde{S}}_{e}\left(t\right),0,0,\frac{\pi }{\mu }-{\tilde{S}}_{e}\left(t\right),\frac{\pi }{\mu }\right)$, where*

(6) $$\left\{\begin{array}{c} \begin{array}{cc} {\tilde{S}}_{e}\left(t\right)=\frac{\pi }{\mu }+\left(S^{*}-\frac{\pi }{\mu }\right)e^{-\mu (t-nT)}, & nT<t⩽(n+1)T,\\ S^{*}=\frac{\pi \left(1-\varphi \right)\left(1-e^{-\mu T}\right)}{\mu \left[1-\left(1-\varphi \right)e^{-\mu T}\right]}, & t=nT. \end{array} \end{array}\right.$$

**Remark** **3.** *The proof process follows a similar approach to that described in [21,22,36], which will be omitted in this context.*

## 4. Global Attractivity of Rumor-Free Periodic Solution

**Definition** **1** ([37]). *The rumor-free periodic solution for the system in Equation *(3)* is considered globally attractive if for any t⩾0, $\lim_{t\to \infty }\left(S(t),I(t),D(t),R(t),N(t)\right)=\left({\tilde{S}}_{e}\left(t\right),0,0,\frac{\pi }{\mu }-{\tilde{S}}_{e}\left(t\right),\frac{\pi }{\mu }\right).$*

As is common knowledge, the basic reproduction number plays a crucial role as a critical threshold in determining the spread of rumors. In this section, we will derive the basic reproduction number and provide evidence that the rumor-free periodic solution is globally attractive when the basic reproduction number $R_{1}$ is less than unity.

For the system in Equation (3), we define the basic reproduction number

(7) $$\begin{array}{c} R_{1}=\frac{\alpha e^{-\mu \tau }B}{\left(1+\rho B\right)\sigma },\; \end{array}$$

where $\alpha =\max\left\{\alpha_{1},\alpha_{2}\right\},\rho =\min\left\{\rho_{1},\rho_{2}\right\},\;B=\frac{\pi \left(1-e^{-\mu T}\right)}{\mu \left[1-\left(1-\varphi \right)e^{-\mu T}\right]}$, and $\sigma =\min\left\{\theta \gamma_{1}+\mu ,\gamma_{2}+\mu \right\}.$

**Theorem** **1.** *When $R_{1}<1$, then the rumor-free periodic solution for the system in Equation *(3)* is globally attractive.*

**Proof.**  Since$\;R_{1}<1$, a sufficiently small ε>0 can be chosen such that

(8) $$\begin{array}{cc} \; & \frac{\alpha e^{-\mu \tau }\left(B+\varepsilon \right)}{1+\rho (B+\varepsilon )}<\sigma ,\;\alpha =\max\left\{\alpha_{1},\;\alpha_{2}\right\},\\ \; & \rho =\min\left\{\rho_{1},\;\rho_{2}\right\},\;B=\frac{\pi \left(1-e^{-\mu T}\right)}{\mu \left[1-\left(1-\varphi \right)e^{-\mu T}\right]},\\ \; & \sigma =\min\left\{\theta \gamma_{1}+\mu ,\;\gamma_{2}+\mu \right\}. \end{array}$$

Based on the first and sixth equations in the system in Equation (3), the following can be obtained:

$$\left\{\begin{array}{c} \begin{array}{cc} \frac{dS(t)}{dt}⩽\pi -\mu S\left(t\right), & t\neq nT,\\ S\left(t^{+}\right)=\left(1-\varphi \right)S\left(t\right), & t=nT. \end{array} \end{array}\right.$$

Then, consider the comparison impulsive differential system:

(9) $$\left\{\begin{array}{c} \begin{array}{ccc} \frac{dx(t)}{dt} & =\pi -\mu x(t), & t\neq nT,\\ x\left(t^{+}\right) & =(1-\varphi )x(t), & t=nT.\; \end{array} \end{array}\right.$$

It is widely recognized that the system in Equation (9) possesses a global asymptotically stable positive periodic solution ${\tilde{x}}_{e}\left(t\right)$, and it has

$${\tilde{x}}_{e}\left(t\right)=\frac{\pi }{\mu }+\left(x^{*}-\frac{\pi }{\mu }\right)e^{-\mu (t-nT)},\;nT<t\leq \left(n+1\right)T,$$

where

$$x^{*}=\frac{\pi \left(1-\varphi \right)\left(1-e^{-\mu T}\right)}{\mu \left[1-\left(1-\varphi \right)e^{-\mu T}\right]}.$$

Under the comparison theorem [38], there exists a positive integer $l_{1}>0$ such that

$$\begin{array}{cc} \; & S\left(t\right)<x\left(t\right)<{\tilde{x}}_{e}\left(t\right)+\varepsilon ,\;nT<t⩽\left(n+1\right)T,\;n>l_{1},\\ \; & S\left(t\right)<{\tilde{x}}_{e}\left(t\right)+\varepsilon \leq \frac{\pi \left(1-e^{-\mu T}\right)}{\mu \left[1-\left(1-\varphi \right)e^{-\mu T}\right]}+\varepsilon =\eta . \end{array}$$

Next, according to the system in Equation (3), it can be inferred that when t>nT+τ, and $n>l_{1}$, there is

$$\frac{d}{dt}\left(I(t)+D(t)\right)⩽\frac{\alpha \eta e^{-\mu \tau }}{1+\rho \eta }\left(I\left(t-\tau \right)+D\left(t-\tau \right)\right)-\sigma \left(I\left(t\right)+D\left(t\right)\right).$$

Then, consider the comparison differential systems:

(10) $$\frac{dy(t)}{dt}=\frac{\alpha \eta e^{-\mu \tau }}{1+\rho \eta }y\left(t-\tau \right)-\sigma y\left(t\right).$$

From Equation (8), we can see that $\frac{\alpha \eta e^{-\mu \tau }}{1+\rho \eta }<\sigma .$ By applying Lemma 1 in [36], it can be deduced that $\lim_{t\to \infty }y\left(t\right)=0.$

Based on the comparison theorem for differential equations, and taking into consideration that I(t)⩾0and D(t)⩾0, it can be inferred that $\lim_{t\to \infty }\left(I\left(t\right)+D\left(t\right)\right)=0$. Hence, it can be concluded that $\lim_{t\to \infty }I\left(t\right)=\lim_{t\to \infty }D\left(t\right)=0$. In summation, we have

(11) $$\left\{\begin{array}{c} \begin{array}{c} I(t)⩾0,\\ D(t)⩾0,\\ \lim_{t\to \infty }I\left(t\right)=\lim_{t\to \infty }D\left(t\right)=0. \end{array} \end{array}\right.$$

According to Equation (11), it can be found that for any sufficiently small $\varepsilon_{1}>0$, there exists an integer $l_{2}$, and $l_{2}T>l_{1}T+\tau$ such that

(12) $$0<I\left(t\right),\;D\left(t\right)<\varepsilon_{1},\;\forall t>l_{2}T.\;$$

Since $\lim_{t\to \infty }N\left(t\right)=\frac{\pi }{\mu },$ it follows that there exists an integer $l_{3}$ that satisfies $l_{3}>l_{2}$ and ensures that

$$0<I\left(t\right),\;D\left(t\right)<\varepsilon_{1},\;N\left(t\right)>\frac{\pi }{u}-\varepsilon_{1},\forall t>l_{3}T.$$

Additionally, it follows from the second equation of the system in Equation (1) that

$$\begin{array}{cc} \frac{dH(t)}{dt} & ⩽\alpha \left(\frac{S(t)I(t)}{1+\rho_{1}S\left(t\right)}+\frac{S(t)D(t)}{1+\rho_{2}S\left(t\right)}\right)-\mu H\left(t\right)\\ \; & \leq \alpha \varepsilon_{1}\left(\frac{S(t)}{1+\rho_{1}S\left(t\right)}+\frac{S(t)}{1+\rho_{2}S\left(t\right)}\right)-\mu H\left(t\right)\\ \; & \leq 2\alpha \varepsilon_{1}\frac{S(t)}{1+\rho S(t)}-\mu H\left(t\right)\\ \; & \leq 2\alpha \varepsilon_{1}\frac{\frac{\pi }{\mu }}{1+\rho \frac{\pi }{\mu }}-\mu H\left(t\right)\\ \; & =2\alpha \varepsilon_{1}\frac{\pi }{\mu +\rho \pi }-\mu H\left(t\right),\;\forall t>l_{3}T. \end{array}$$

We know that there exists an integer $l_{4}>l_{3}$ such that

$$H\left(t\right)<\frac{2\alpha \varepsilon_{1}\pi }{\mu (\mu +\rho \pi )}+\varepsilon_{1},\;\forall t>l_{4}T.$$

Let $W\left(t\right)=\left|S\left(t\right)-\tilde{S}e\left(t\right)\right|$. If there is a positive integer $l_{5}>l_{4}$, then

$$\begin{array}{cc} D^{+}W\left(t\right) & ⩽-\mu W\left(t\right)+\alpha_{1}\frac{S(t)I(t)}{1+\rho_{1}S\left(t\right)}+\alpha_{2}\frac{S(t)D(t)}{1+\rho_{2}S\left(t\right)}\\ \; & ⩽P\varepsilon_{1}-\mu W\left(t\right),\;\forall t>l_{5}T,\; \end{array}$$

where $P=\frac{2\alpha \pi }{\mu +\rho \pi }$ and$\;W\left(t^{+}\right)=\left(1-\varphi \right)W\left(t\right)\;\mathrm{when}\;t=nT$.

Consider the following differential equation:

$$\frac{dw(t)}{dt}=P\varepsilon_{1}-\mu w\left(t\right).$$

We know that $0⩽W\left(t\right)⩽w\left(t\right),\;\forall t>l_{5}T$ and

$$\lim_{t\to \infty }w\left(t\right)=\frac{P\varepsilon_{1}}{\mu }.$$

Here is a positive integer $l_{6}>l_{5}$ such that

$$0⩽W\left(t\right)⩽\frac{P\varepsilon_{1}}{\mu }+\varepsilon_{1},\;\forall t>l_{6}T.$$

Assuming that $\varepsilon_{1}>0$ is sufficiently small, the equation above simplifies to

$$\lim_{t\to \infty }H\left(t\right)=0,\;\lim_{t\to \infty }S\left(t\right)={\tilde{S}}_{e}\left(t\right).$$

Based on the equation N(t)=S(t)+H(t)+I(t)+D(t)+R(t), it can be concluded that $\lim_{t\to \infty }R\left(t\right)=\frac{\pi }{\mu }-{\tilde{S}}_{e}\left(t\right).$

In conclusion, the rumor-free periodic solution $\left({\tilde{S}}_{e}\left(t\right),0,0,\frac{\pi }{\mu }-{\tilde{S}}_{e}\left(t\right),\frac{\pi }{\mu }\right)$ for the system in Equation (3) is globally attractive if $R_{1}<1$. □

**Remark** **4.** *When $R_{1}<1$, the densities of the susceptible individuals and the refuting individuals gradually approach zero over time, effectively controlling the spread of the rumor.*

**Corollary** **1.** *The rumor-free periodic solution $\left({\tilde{S}}_{e}\left(t\right),0,0,\frac{\pi }{\mu }-{\tilde{S}}_{e}\left(t\right),\frac{\pi }{\mu }\right)$ for the system in Equation *(3) is globally attractive if $\varphi >\varphi^{*}$, where

$$\left\{\begin{array}{c} \begin{array}{c} \varphi^{*}=\left(e^{-\mu T}-1\right)\left[\frac{\pi }{\mu }\left(\frac{\alpha e^{-\mu \tau }}{\sigma }-\rho \right)-1\right],\\ \alpha =\max\left\{\alpha_{1},\;\alpha_{2}\right\},\\ \sigma =\min\left\{\theta \gamma_{1}+\mu ,\;\gamma_{2}+\mu \right\}. \end{array} \end{array}\right.$$

**Proof.**  Let $\varphi =\varphi^{*}$ in $R_{1}$ make $R_{1}\left(\varphi^{*}\right)=1$, which is the value that replaces φ of $R_{1}$ with $\varphi^{*}$, resulting in the following:

$$\left\{\begin{array}{c} \begin{array}{c} \varphi^{*}=\left(e^{-\mu T}-1\right)\left[\frac{\pi }{\mu }\left(\frac{\alpha e^{-\mu \tau }}{\sigma }-\rho \right)-1\right],\;\\ \alpha =\max\left\{\alpha_{1},\;\alpha_{2}\right\},\\ \sigma =\min\left\{\theta \gamma_{1}+\mu ,\;\gamma_{2}+\mu \right\}. \end{array} \end{array}\right.$$

By taking the derivative of $R_{1}$ with respect to φ, we can obtain

$$\frac{dR_{1}}{d\varphi }=\alpha e^{-\mu \tau }\frac{dB}{d\varphi }\Rightarrow \frac{dB}{d\varphi }=\frac{-\pi \left(1-e^{-\mu T}\right)e^{-\mu T}}{\mu {\left[1-\left(1-\varphi \right)e^{-\mu T}\right]}^{2}}<0.$$

Hence, we have

(13) $$\frac{dR_{1}}{d\varphi }<0.$$

Since $R_{1}$ is monotonically decreasing with respect to φ, the conclusion logically follows:

$$\mathrm{if}\;\varphi >\varphi^{*},\;\mathrm{then}\;R_{1}<1.$$

According to Theorem 1, the rumor-free periodic solution $\left({\tilde{S}}_{e}\left(t\right),0,0,\frac{\pi }{\mu }-{\tilde{S}}_{e}\left(t\right),\frac{\pi }{\mu }\right)$ for the system in Equation (3) is globally attractive. □

## 5. Permanence

This section delves into the notion of permanence in the rumor-spreading process, which refers to the infected individuals I(t) and the refuting individuals D(t) in the system in Equation (3) remaining present and active over time, with a certain proportion still existing in the system.

**Definition** **2** ([39]). *We define that in the system in Equation *(3)*, the rumor is considered to be permanent if there exists a positive constant m such that ${lim inf}_{t\to \infty }I\left(t\right)+D\left(t\right)⩾m$ for any positive solution (S(t),I(t),D(t),R(t),N(t)) for the system in Equation *(3)* with the initial condition in Equation *(4).

For the system in Equation (3), the basic reproduction number $R_{2}$ is defined as follows:

(14) $$R_{2}=\left(\frac{\alpha^{\prime }e^{-\mu \tau }}{\sigma^{\prime }}-\rho^{\prime }\right)\frac{\pi \left(1-\varphi \right)\left(1-e^{-\mu T}\right)}{\mu \left[1-\left(1-\varphi \right)e^{-\mu T}\right]},$$

where $\alpha^{\prime }=\min\left\{\alpha_{1},\alpha_{2}\right\},\;\rho^{\prime }=\max\left\{\rho_{1},\rho_{2}\right\}$, and $\sigma^{\prime }=\max\left\{\theta \gamma_{1}+\mu ,\gamma_{2}+\mu \right\}.$

**Theorem** **2.** *If $R_{2}>1$, then the rumor is permanent.*

**Proof.**  According to the system in Equation (3), if we let F(t)=I(t)+D(t), then we have

(15) $$\begin{array}{cc} \frac{dF(t)}{dt} & ⩾\frac{\alpha^{\prime }e^{-\mu \tau }}{1+\rho^{\prime }S\left(t-\tau \right)}S\left(t-\tau \right)F\left(t-\tau \right)-\sigma^{\prime }F\left(t\right)\\ \; & =F\left(t\right)\left(\frac{\alpha^{\prime }e^{-\mu \tau }S\left(t\right)}{1+\rho^{\prime }S\left(t\right)}-\sigma^{\prime }\right)-\alpha^{\prime }e^{-\mu \tau }\frac{d}{dt}\int_{t-\tau }^{t}\frac{S(u)F(u)}{1+\rho^{\prime }S\left(u\right)}du. \end{array}$$

Let

(16) $$V\left(t\right)=F\left(t\right)+\alpha^{\prime }e^{-\mu \tau }\int_{t-\tau }^{t}\frac{S(u)F(u)}{1+\rho^{\prime }S\left(u\right)}du.$$

The derivative of Equation (16) can be obtained as follows:

(17) $$\begin{array}{cc} \frac{dV(t)}{dt} & ⩾F\left(t\right)\left(\frac{\alpha^{\prime }e^{-\mu \tau }S\left(t\right)}{1+\rho^{\prime }s\left(t\right)}-\sigma^{\prime }\right)\\ \; & =\sigma^{\prime }F\left(t\right)\left(\frac{\alpha^{\prime }e^{-\mu \tau }S\left(t\right)}{\sigma^{\prime }\left(1+\rho^{\prime }S\left(t\right)\right)}-1\right). \end{array}$$

If we define $F^{*}=\frac{\mu }{\alpha }\left(R_{2}-1\right)$, then the following statements hold true when ε is sufficiently small:

(1) If $R_{2}>1$, then $F^{*}>0$, and (2) when $\varepsilon =R_{2}-1\to 0^{+}$, $F^{*}\to 0^{+}$$\Rightarrow \frac{\alpha^{\prime }e^{-\mu \tau }\eta^{\prime }}{\sigma^{\prime }\left(1+\rho^{\prime }\eta^{\prime }\right)}>1,$ where

$$\eta^{\prime }=\frac{\pi \left(1-\varphi \right)\left(1-e^{-\left(\alpha F^{*}+\mu \right)T}\right)}{\left(\alpha F^{*}+\mu \right)\left[1-\left(1-\varphi \right)e^{-\left(\alpha F^{*}+\mu \right)T}\right]}-\varepsilon >0.\;$$

Assuming that for any $t_{1}>0$, $F\left(t\right)<F^{*}$ is impossible for all $t⩾t_{1}$, if it contradicts the assumption, then there exists $t_{1}>0$ such that $F\left(t\right)<F^{*}$ for all $t⩾t_{1}$.

From the first and sixth equations of the system in Equation (3), we can conclude that for any $t⩾t_{1}$, we have

(18) $$\left\{\begin{array}{c} \begin{array}{ccc} \frac{dS(t)}{dt} & ⩾\pi -\frac{\alpha S(t)}{1+\rho S(t)}F\left(t\right)-\mu S\left(t\right)\\ \; & >\pi -\alpha S\left(t\right)F^{*}-\mu S\left(t\right)\\ \; & =\pi -\left(\alpha F^{*}+\mu \right)S\left(t\right), & t\neq nT,\;\\ S\left(t^{+}\right) & =(1-\varphi )S(t), & t=nT.\; \end{array} \end{array}\right.$$

where$\;\alpha =\max\left\{\alpha_{1},\alpha_{2}\right\}$.

If we consider the following comparison impulsive differential system for all $t⩾t_{1}$, then one has

(19) $$\left\{\begin{array}{c} \begin{array}{ccc} \frac{dx_{1}\left(t\right)}{dt} & =\pi -\left(\alpha F^{*}+\mu \right)x_{1}\left(t\right), & t\neq nT,\\ x_{1}\left(t^{+}\right) & =\left(1-\varphi \right)x_{1}\left(t\right), & t=nT. \end{array} \end{array}\right.$$

The periodic solution for the system in Equation (18) is globally asymptotically stable, which can be expressed as

$$\begin{array}{cc} \; & {\tilde{x}}_{1e}\left(t\right)=\frac{\pi }{\alpha F^{*}+\mu }+\left(x_{1}^{*}-\frac{\pi }{\left.\alpha F^{*}+\mu \right.}\right)e^{\left.-\left(\alpha F^{*}+\mu \right)\left(t-nT\right)\right.},\;nT<t⩽\left(n+1\right)T,\; \end{array}$$

where

$$\begin{array}{cc} \; & x_{1}^{*}=\frac{\pi \left(1-\varphi \right)\left(1-e^{-\left(\alpha F^{*}+\mu \right)T}\right)}{\left(\alpha F^{*}+\mu \right)\left[1-\left(1-\varphi \right)e^{\left.-\left(\alpha F^{*}+\mu \right)T\right]}\right.}\;. \end{array}$$

Based on the comparison theorem for the impulsive differential equation, it can be concluded that there exists $t_{2}>t_{1}+\tau \;$such that $S\left(t\right)>{\tilde{x}}_{1e}\left(t\right)-\varepsilon$, $\forall t⩾t_{2}$, and thus

(20) $$S\left(t\right)>x_{1}^{*}-\varepsilon =\eta^{\prime },\;\forall t⩾t_{2}.$$

If we let $F_{1}=\min_{t\in [t_{2},t_{2}+\tau ]}F\left(t\right)$, then it follows that $F\left(t\right)⩾F_{1}$ for an arbitrary $t⩾t_{2}.$ Otherwise, there then exists $T_{0}>0$ such that $F\left(t\right)⩾F_{1}\;\mathrm{holds}\;\mathrm{for}\;\forall t\in \left[t_{2},t_{2}+\tau +T_{0}\right]$ and $F\left(t_{2}+\tau +T_{0}\right)=F_{1},\;F^{\prime }\left(t_{2}+\tau +T_{0}\right)\leq 0.\;$However, it can be known from the system in Equations (3) and (20) that

$$\begin{array}{cc} F^{\prime }\left(t_{2}+\tau +T_{0}\right) & ⩾\alpha^{\prime }e^{-\mu \tau }\frac{S\left(t_{2}+T_{0}\right)F\left(t_{2}+T_{0}\right)}{1+\rho^{\prime }S\left(t_{2}+T_{0}\right)}-\sigma^{\prime }F\left(t_{2}+T_{0}+\tau \right)\\ \; & ⩾\sigma^{\prime }F_{1}\left(\frac{\alpha^{\prime }e^{-\mu \tau }\eta^{\prime }}{\sigma^{\prime }\left(1+\rho^{\prime }\eta^{\prime }\right)}-1\right)\\ \; & >0, \end{array}$$

This is a contradiction to $F^{\prime }\left(t_{2}+\tau +T_{0}\right)\leq 0$. Thus, $F\left(t\right)⩾F_{1}$ for an arbitrary $t⩾t_{2}$.

Then, it can be further seen from Equations (17) and (20) that

(21) $$V^{\prime }\left(t\right)>\sigma^{\prime }F_{1}\left(\frac{\alpha^{\prime }e^{-\mu \tau }\eta \prime }{\sigma^{\prime }\left(1+\rho^{\prime }\eta \prime \right)}-1\right)>0.$$

This means that for all $t⩾t_{2},\;\mathrm{if}\;t\to \infty$, then V(t)→∞.

However, there is also

$$\begin{array}{cc} V(t) & =F\left(t\right)+\alpha^{\prime }e^{-\mu \tau }\int_{t-\tau }^{t}\frac{S(u)F(u)}{1+\rho^{\prime }S\left(u\right)}du\\ \; & ⩽F\left(t\right)+\alpha^{\prime }e^{-\mu \tau }\int_{t-\tau }^{t}S\left(u\right)F\left(u\right)du\\ \; & ⩽\frac{\pi }{\mu }+\alpha^{\prime }e^{-\mu \tau }\int_{t-\tau }^{t}{\left(\frac{\pi }{\mu }\right)}^{2}du=\frac{\pi }{\mu }\left(1+\frac{\pi \tau \alpha^{\prime }e^{-\mu \tau }}{\mu }\right),\; \end{array}$$

This is in contradiction with Equation (21).

Therefore, the assumption is true, and we can draw the conclusion that it is not possible for F(t) to be consistently less than $F^{*}$ for all $t_{1}>0$. Therefore, there are two possible scenarios to consider: (1) $F\left(t\right)⩾F^{*}$ when *t* is sufficiently large, and (2) F(t) oscillates around $F^{*}$ when *t* is sufficiently large.

The parameters are given as

(22) $$P=\min\left\{\frac{F^{*}}{2},\;P_{1}\right\},$$

where $P_{1}=F^{*}e^{-\sigma^{\prime }\tau }$ and$\;\sigma^{\prime }=\max\left\{\theta \gamma_{1}+\mu ,\;\gamma_{2}+\mu \right\}$.

Our objective is to demonstrate that F(t)⩾P for sufficiently large values of *t*. While the first case has already been established, we will now shift our focus to the second case.

Let $t_{0}>0\;$andq>0 satisfy $F\left(t_{0}\right)=F\left(t_{0}+q\right)=F^{*}$ and $\;F\left(t\right)<F^{*}$ for $t_{0}<t<t_{0}+q$, where $t_{0}$ is sufficiently large such that S(t)>η′ for $t_{0}<t<t_{0}+q$.

The solution for the system in Equation (3) is positive and ultimately bounded, and the impulsive vaccination does not have an effect on the infected individuals or the refuting individuals. As a result, F(t) is uniformly continuous.

Therefore, there is a constant $T^{\prime }\left(0<T^{\prime }<\tau \right.$, and $T^{\prime }$ is independent of $t_{0}$) such that for any $t_{0}⩽t⩽t_{0}+T^{\prime }$, there exists $F\left(t\right)>\frac{F^{*}}{2}$. If $q⩽T^{\prime }$, then the result we need is already contained. If $T^{\prime }<q⩽\tau$, then since $F^{\prime }\left(t\right)>-\sigma^{\prime }F\left(t\right)$ and $F\left(t_{0}\right)=F^{*}$, it is obvious that for $t_{0}<t<t_{0}+q$, we have $F\left(t\right)⩾P_{1}$. If q>τ, then for $t_{0}<t<t_{0}+\tau$, we have $F\left(t\right)⩾P_{1}$. Based on the proof of the above part, it is known that for $t_{0}+\tau <t<t_{0}+q$, we have $F\left(t\right)⩾P_{1}$. As the interval $\left[t_{0},t_{0}+\tau \right]$ is random, we can conclude that in the second case, F(t)⩾P is satisfied if *t* is sufficiently large.

Based on the analysis conducted, it can be concluded that the selection of *P* is not related to the positive solutions for the system in Equation (3). Additionally, it has been proven that when *t* is large enough, all positive solutions for the system in Equation (3) meet the requirement of F(t)≥P. This completes the proof of Theorem 2. □

**Remark** **5.** *In contrast to [21,22], when rumors persist despite the presence of refuting individuals and susceptible individuals, not only do the susceptible individuals persist, but the refuting individuals also persist in their efforts to counteract the spread of the rumor.*

## 6. Numerical Simulation

In this section, some simulation examples are put forward to testify to the validity of the results. For the sake of convenience, the initial value of the system in Equation (1) was selected follows: S(0) = 0.92, H(0) = 0.03, I(0) = 0.02, D(0) = 0.02, and R(0) = 0.01.

### 6.1. Effect of the Impulsive Vaccination Rate φ on the System

**Figure 2:**
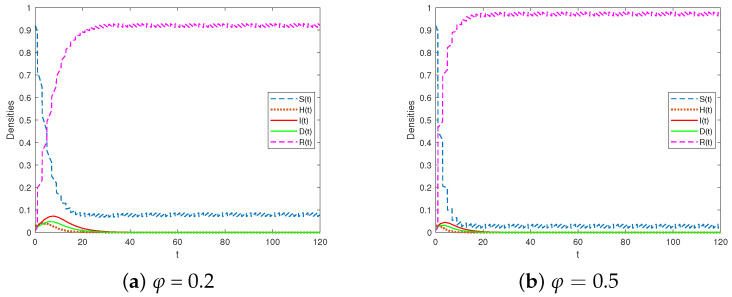
The rumor-free periodic solution is globally attractive for $R_{1}<1$.

The selection parameters are shown in Scheme 1 of Table 2. We chose T=2 and τ=1. By considering the impulsive vaccination proportions of φ at 0.2 and 0.5 (as shown in Figure 2), it could be calculated that $R_{1}<1$. The results depicted in Figure 2 demonstrate that the rumor-free periodic solution for the system in Equation (3) is globally attractive. This indicates that the densities of H(t), I(t), and D(t) decreased over time, signifying effective control of the rumor. Furthermore, the graphs illustrate that increasing the impulsive vaccination proportion φ led to a significant decrease in the proportion of susceptible individuals. Corollary 1 confirms that a high impulsive vaccination proportion can guarantee the global attractiveness of the rumor-free periodic solution.

**Figure 3:**
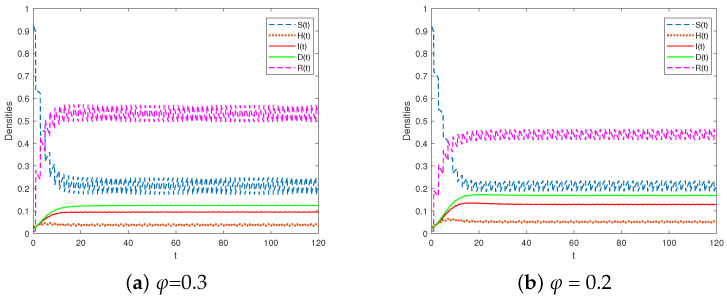
The system in Equation (3) is permanent for $R_{2}>1$.

The selection parameters are presented in Scheme 2 of Table 2. We chose T=2 and τ=1. Upon examining the impulsive vaccination proportions of φ at 0.2 and 0.3 (as depicted in Figure 3), it was observed that $R_{2}>1$, indicating the persistence of the system. As the impulsive vaccination rate φ decreased, a higher proportion of densities of I(t) and D(t) was observed, suggesting that small impulsive vaccination can contribute to the spread of rumors. Increasing the impulsive vaccination rate effectively controlled rumors. Consequently, the findings suggest the need to enhance popular science education in practical settings to mitigate the propagation of misinformation.

### 6.2. Effect of the Impulsive Period *T* on the System

**Figure 4:**
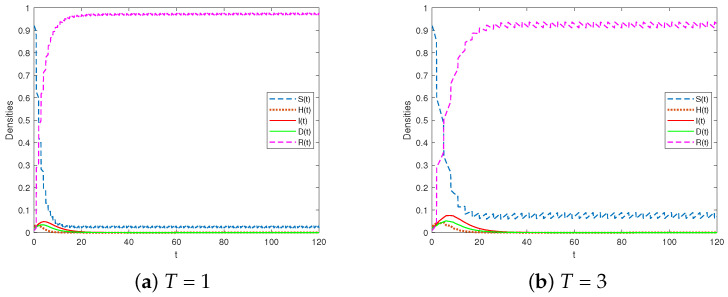
The rumor-free periodic solution is globally attractive for $R_{1}<1$.

Considering the cases of T=1 or T=3, we selected the parameters as shown in Scheme 1. Additionally, we selected φ=0.3 and τ=1. Our calculations show that $R_{1}<1$, and Figure 4 illustrates the evolutionary trends of different categories in the system in Equation (1). The findings indicate that as the impulsive period decreased, there was a decrease in the proportion of susceptible individuals. Additionally, an increase in the impulsive period *T* led to a higher peak value of infected individuals and a longer duration for the rumor to dissipate.

**Figure 5:**
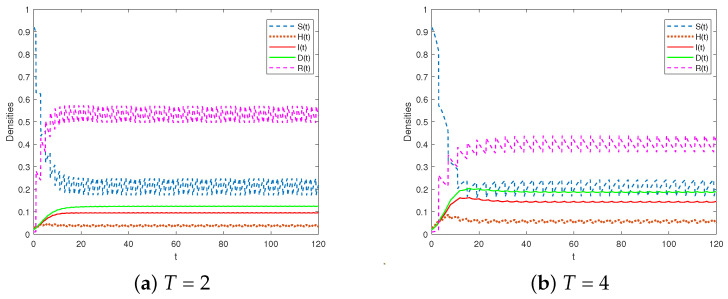
The system in Equation (3) is permanent for $R_{2}>1$.

Considering the cases of T=2 or T=4, we selected the parameters as shown in Scheme 2. Additionally, we selected φ=0.3 and τ=1. We calculated the persistence of the system in Equation (3). Our findings indicate that $R_{2}>1$, indicating the persistence of the system. Furthermore, Figure 5 illustrates that as the impulsive period increased, there was an increase in the proportion of infected individuals and refuting individuals. In order to effectively control rumors, it is crucial to reduce the impulsive period *T* and enhance the frequency of popular science education.

### 6.3. Effect of the Time Delay τ on the System

**Figure 6:**
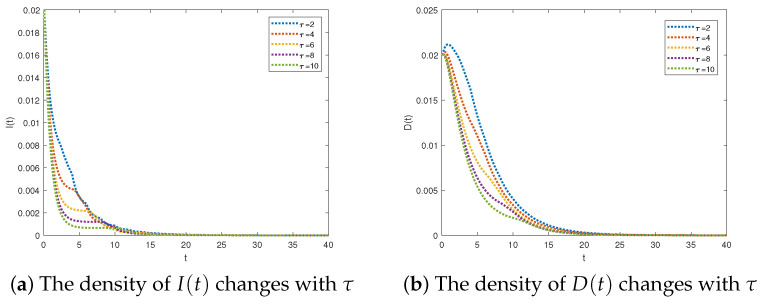
The rumor-free periodic solution is globally attractive for $R_{1}<1$.

Taking into account the scenarios where τ=2,4,6,8,10, we opted for the parameters outlined in Scheme 3. Additionally, we selected φ=0.3 and T=2. It can be demonstrated that $R_{1}<1$. The study depicted in Figure 6 demonstrates that the system is globally attractive, indicating that the rumor will eventually disappear. Furthermore, the findings suggest that as τ decreased, there was a slower decrease in the proportion of infected individuals. This can be attributed to the fact that individuals have less time to process and release accurate information. As a result, more individuals believe and spread the rumor simultaneously, leading to a larger proportion of infected individuals.

**Figure 7:**
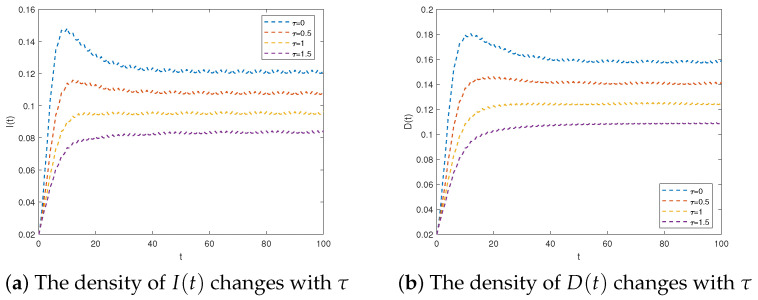
The system in Equation (3) is permanent for $R_{2}>1$.

Taking into account the scenarios where τ=0.5,1,1.5, we opted for the parameters outlined in Scheme 2. Furthermore, by selecting φ = 0.3 and *T* = 2, we achieved $R_{2}>1$, indicating a persistent system. The results depicted in Figure 7 illustrate that increasing the time delay can have a significant impact on reducing the maximum number of infected individuals and refuting individuals, thereby limiting the spread of rumors. This emphasizes the importance of exercising critical thinking and caution when evaluating information. Allowing more time to discern the validity of information can effectively minimize the harmful effects of rumors.

### 6.4. Effect of $\rho_{1}$ and $\rho_{2}$ on the System

To facilitate the analysis, we chose parameter values of $\rho_{1}$ = $\rho_{2}$ = 0, 0.05, 0.2, 0.4, 0.6, 0.8 while keeping the other parameters fixed, as depicted in Figure 2a.

**Figure 8:**
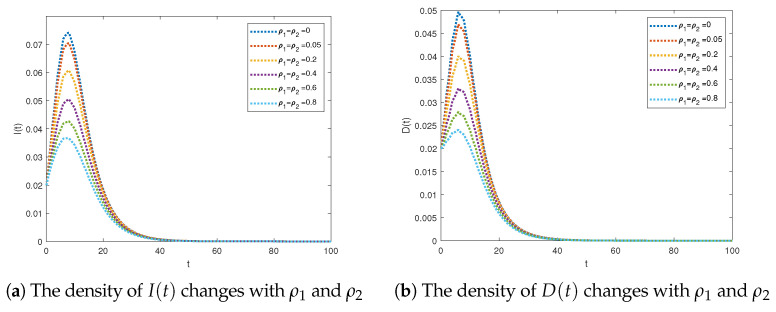
The rumor-free periodic solution is globally attractive for $R_{1}<1$.

As illustrated in Figure 8, the densities of I(t) and D(t) exhibited a gradual decrease as $\rho_{1}$ (or $\rho_{2}$) increased, eventually converging to zero. The observation that an increase in psychological factors results in a decrease in the spread of rumors suggests that incorporating the nonlinear incidence aligns with real-world scenarios.

### 6.5. The Relationship between the Basic Reproduction Number and the Parameter Value

**Figure 9:**
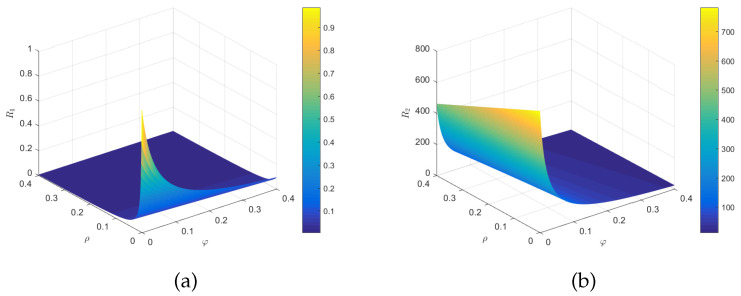
(**a**) The relationship of $R_{1}$ with parameters ρ and φ. (**b**) The relationship of $R_{2}$ with parameters ρ and φ.

Let us examine the relationship between $R_{1}$ and $R_{2}$ with respect to the parameters ρ and φ. We chose the variables φ∈ [0, 0.4], ρ∈ [0, 0.4]. From Figure 9, it is evident that both $R_{1}$ and $R_{2}$ decreased as ρ and φ increased. This implies that enhancing the impact of psychological factors and increasing the proportion of impulsive vaccination can effectively reduce the threshold for rumor outbreaks, thereby suppressing the rumor-spreading.

## 7. Conclusions

This paper presents the SHIDR rumor model with nonlinear incidence, refuting individuals, impulsive vaccination, and time delays. The comparison principle of impulsive differential systems was applied to obtain two thresholds, namely $R_{1}$ and $R_{2}$. For $R_{1}<1$, the global attractiveness of the rumor-free periodic solution was explored, indicating that the rumor would eventually disappear. Furthermore, the numerical simulation results suggest that increasing the proportion of impulsive vaccination, reducing the impulsive period, or increasing the time delay can facilitate the eventual disappearance of the rumor. Conversely, when $R_{2}>1$, the system remained persistent, indicating the continued existence of the rumor. The findings of this study demonstrate that decreasing impulsive vaccination, extending the impulsive period, or reducing the time delay exacerbates the spread of the rumor. In the context of OSNs, there are several practical factors that should still be taken into consideration when modeling rumor propagation. These factors include the randomness of rumor propagation and the occurrence of Hopf bifurcation due to time delays. These are the areas that we will focus on in our future research endeavors. 

## Figures and Tables

**Table 1:** Parameters and meanings.

Parameter	Meaning
π	Rate of susceptible individuals joining the network
μ	Rate at which individuals leave the network
$\gamma_{1}$	Probability of infected individuals becoming refuting individuals
$\alpha_{1}$	Propagation rate of contact between susceptible and infected individuals
$\alpha_{2}$	Propagation rate of contact between susceptible and refuting individuals
$\rho_{1}$	Psychological influence factor for the spread of rumors
$\rho_{2}$	Psychological influence factor for the spread of the truth
$\gamma_{2}$	Probability of refuting individuals becoming recovered individuals
*k*	Proportion at which hesitant individuals turn into infected individuals
φ	The rate of the *n*th impulsive vaccination of the susceptible individuals

**Table 2:** Values of the parameters.

Parameter	$\alpha_{1}$	$\alpha_{2}$	$\rho_{1}$	$\rho_{2}$	$\gamma_{1}$	$\gamma_{2}$	θ	κ	μ	π
Scheme 1	0.8	0.9	0.02	0.03	0.2	0.5	0.5	0.5	0.01	0.01
Scheme 2	0.8	0.9	0.02	0.03	0.09	0.08	0.5	0.5	0.1	0.1
Scheme 3	0.8	0.4	0.08	0.1	0.8	0.1	0.5	0.7	0.3	0.3

## Data Availability

Data are contained within the article.
